# General anesthesia with propofol for ovarian teratoma excision associated with anti-N-methyl-D-aspartate receptor encephalitis

**DOI:** 10.1186/s40981-018-0153-6

**Published:** 2018-02-02

**Authors:** Masami Sato, Hiroaki Yasumoto, Toshiyuki Arai

**Affiliations:** 1 Department of Anesthesia, Kyoto City Hospital, 1-2 Mibuhigashitakada-cho, Nakagyo-ku, Kyoto, 604-8845 Japan; 2 Department of Anesthesia and Critical Care Medicine, Kyoto City Hospital, Kyoto, Japan

**Keywords:** Anti-N-methyl-D-aspartate receptor encephalitis, Ovarian teratoma, Propofol

## Abstract

Anti-N-methyl-D-aspartate receptor (NMDAR) encephalitis is an autoimmune disorder caused by production of anti-NMDAR antibodies that is often associated with ovarian teratoma and exhibits various manifestations including psychiatric symptoms, seizures, hypoventilation, and autonomic nerve instability. Patients with this disorder who receive early surgical tumor resection along with immunotherapy have better outcome than the rest of the patients. To establish an anesthetic plan, it is important to understand the pharmacological interaction between the anesthetic agents and the disabled NMDAR, because NMDAR is one of the major sites of action for commonly-used anesthetic agents. Herein, we describe two young female patients with anti-NMDAR encephalitis who required surgical resection of ovarian teratoma under general anesthesia using propofol, remifentanil, and fentanyl. In both of these anesthetic courses, neither psychoneuronal modification nor autonomic instability by propofol was evident. Furthermore, propofol has been reported to suppress the effects of ketamine on the posterior cingulate cortices, which is the area of the brain concerned with psychotomimetic activity and neural damage of NMDAR antagonists. Our cases imply that propofol is safely used in patients with anti-NMDAR encephalitis, although it has some pharmacological effects on NMDAR.

## Background

Anti-N-methyl-D-aspartate receptor (NMDAR) encephalitis is an autoimmune neurological disorder caused by production of antibodies to NMDAR and has become the most common autoimmune encephalitis described, since its first description in 2007 [1]. Tumors occur in approximately 38–58% of all cases and 94% of the tumor is ovarian teratoma [2]. Both autoimmune responses against neural antigens expressed ectopically in the underlying tumor and cross-reaction with an unknown infectious agent are thought to be responsible for the pathogenic mechanism of anti-NMDAR encephalitis [1, 2]. The treatment bundle, therefore, includes immunotherapy and surgical tumor resection, if applicable. Patients with early tumor resection along with first-line immunotherapy (corticosteroids, gamma globulin, or plasma exchange) recover earlier, less often require second-line immunotherapy (cyclophosphamide or rituximab, or both) and experience fewer relapses, than those with a tumor that was treated late or without a tumor [3, 4]. To establish an anesthetic plan for patients with anti-NMDAR encephalitis, it is important to understand the pharmacological interaction between the anesthetic agents and the disabled NMDAR, because NMDAR is a major site of action for commonly-used anesthetic agents. Although anti-NMDAR encephalitis has gradually attracted the attention of anesthesiologists, there have been only a few discussions about propofol that have some pharmacological effects on NMDAR.

We describe two cases of young female patients with anti-NMDAR encephalitis who underwent resection of ovarian teratoma under general anesthesia using propofol and discuss the validity of propofol in these patients.

## Case presentation

### Case 1

A 17-year-old woman (159 cm, 62 kg) was admitted to our hospital due to disorientation and aphasia. The patient had suffered from headache and hyperthermia 5 days prior. She was deteriorated with involuntary movements, seizures, paresis of the intestine, and hypersalivation. Tracheal intubation was performed due to aspiration pneumonia and central hypoventilation on day 9 after admission, and mechanical ventilation was started under midazolam sedation. Abdominal computed tomography revealed left ovarian teratoma on the same day. The treatment with gamma globulin and methylprednisolone for anti-NMDAR encephalitis was initiated from day 10 for 3 days. Resection of left ovarian teratoma was scheduled on day 12.

General anesthesia was induced with propofol (target controlled infusion (TCI):3 μg/ml), rocuronium (50 mg) and maintained with oxygen, air, propofol (TCI: 2.5–3 μg/ml), fentanyl (20 μg/h), remifentanil (0.1–0.15 μg/kg/min), and intermittent rocuronium. The case proceeded uneventfully. The patient remained encephalopathic and intubated under midazolam sedation after surgery. Additional immunotherapy with gamma globulin from day 13 after admission for 3 days was ineffective, and the patient presented the clinical manifestations of paroxysmal sympathetic hyperactivity, including increased heart rate and temperature from day 18. The patient suffered from disseminated intravascular coagulation, initiated plasmapheresis from day 25 and underwent tracheostomy on day 37 while in the ICU from day 22 to day 58. After discharge from the ICU, anti-epileptics and high medical care were continued. Rituximab and cyclophosphamide were initiated from day 262 and the encephalitic symptoms were diminished gradually, thereafter. The tracheostomy was closed on day 555. The patient was discharged and advanced to a home rehabilitation program on day 601 after admission.

### Case 2

A 28-year-old woman (159 cm, 45 kg) presented to a mental health clinic with language disorder, short-term memory disturbance, seizure, and hallucination 2 weeks prior. She was transferred to our hospital and abdominal computed tomography revealed right ovarian teratoma. On day 4 after admission, treatment with gamma globulin and methylprednisolone for anti-NMDAR encephalitis was initiated for 3 days. In the following days, the patient developed confusion and was speechless. On day 7, laparoscopic resection of right ovarian teratoma was scheduled.

General anesthesia was induced with propofol (TCI: 6 μg/ml) and fentanyl (100 μg), and tracheal intubation was facilitated with rocuronium (40 mg). Anesthesia was maintained with oxygen, air, propofol (TCI: 2–3 μg/ml), remifentanil (0.1–0.2 μg/kg/min), maintaining bispectral index value of 40–60, and intermittent rocuronium. Bilateral transversus abdominis plane block was performed using 30 ml of 0.375% ropivacaine before surgery. Surgery was completed uneventfully. Rocuronium was reversed with sugamadex (100 mg) and the trachea was extubated. On the night after surgery, she became confused and was treated with oral quetiapine. On the next day after surgery, her neurological status began to improve. After two courses of gamma globulin and methylprednisolone treatment, the patient recovered smoothly and was discharged on day 54 after admission with no neurological symptoms.

## Discussion

The NMDAR is one of two major receptors associated with the effect site of anesthetic agents, along with the gamma-aminobutyric acid type A (GABA_A_) receptor. Ketamine and nitrous oxide minimally affect GABA_A_ receptor and antagonize the NMDAR by direct action [5, 6]. While there have been several reports indicating that propofol also inhibits NMDAR at clinically relevant concentrations, propofol is considered to elicit its anesthetic effects through enhancing a GABAergic mechanism, not via a NMDAR-dependent mechanism [7, 8]. Nakao et al. reported that propofol inhibits ketamine-induced psychotomimetic activities via the activation of GABA_A_ receptor activation in the rat posterior cingulate and retrosplenial cortices, which are suggested to be the regions of brain concerned with psychotomimetic activity and neural damage of NMDAR antagonists [9]. These findings suggest that propofol, a GABAergic agent, is not only effective in suppressing psychotomimetic complications induced by ketamine, but is also acceptable for the suppressing NMDAR-related pathological mechanisms of anti-NMDAR encephalitis.

Several case reports have included the anesthetic procedures using propofol in patients with anti-NMDAR encephalitis. Collectively, Liu et al. and Pascual-Ramírez et al. described three patients who presented for resection of ovarian teratoma under total intravenous anesthesia using propofol. No complications or unexpected events occurred perioperatively in these patients and they recovered smoothly [10, 11]. Pryzbylkowski et al. reported the anesthetic management of two patients and demonstrated that propofol, isoflurane, desflurane, hydromorphone, and fentanyl were well tolerated, and no paroxysmal sympathetic hyperactivity was seen intraoperatively in these cases [12]. On the other hand, Lapebie et al. reported a case of a patient whose symptoms deteriorated with increasing dyskinesia and generalized seizure under propofol sedation after general anesthesia with propofol and sevoflurane for ovarian teratoma resection [13]. There is, however, a limitation to distinguishing complications induced by anesthetic agents from deteriorations of this encephalitis.

In our report, total intravenous anesthesia, including propofol, remifentanil, and fentanyl, was utilized to induce and maintain anesthesia for two patients, and ketamine and nitrous oxide were avoided. In these anesthetic courses, as above, neither psychoneuronal modifications nor autonomic instabilities due to propofol were evident. The first case suffered from prolonged catatonic coma, convulsion, and autonomic instabilities that we treated with using continuous midazolam infusion. The cause of postoperative progress differences in our two cases is suggested that the first case had already more serious symptoms with autonomic nerve instability and central hypoventilation before surgery than the second one.

Although the anesthetic implications of caring for patients with anti-NMDAR encephalitis have not yet been defined, ketamine and nitrous oxide should be avoided because of the uncertainty about the pharmacodynamic response of a disabled NMDAR and benzodiazepines and opiates that do not interfere with the NMDA pathway are preferred in these patients. Propofol acting indirectly at the NMDAR is suggested to be safely used under prudent monitoring and the close titration of dosages judging from NMDAR-related pharmacological mechanism and the past case reports including ours.

## Conclusions

We described two female patients with anti-NMDAR encephalitis who required surgical resection of ovarian teratoma under general anesthesia using propofol. In both of these anesthetic courses, neither psychoneuronal modification nor autonomic instability by propofol was evident. We imply that propofol is safely used in patients with anti-NMDAR encephalitis, although it has some pharmacological effects on NMDAR.

## Abbreviations

NMDAR: N-methyl-D-aspartate receptor
TCI: Target controlled infusion
GABA_A_: Gamma-aminobutyric acid type A
